# Exploring Prejudice Toward Tinder: Two Experiments on the Social Perception of Dating App Users and Online-Formed Couples

**DOI:** 10.3390/bs16050691

**Published:** 2026-04-30

**Authors:** Simona Sciara, Federico Contu, Federica Montano, Carolina Sole Steffano, Giuseppe Pantaleo

**Affiliations:** 1 UniSR-Social.Lab, Faculty of Psychology, Vita-Salute San Raffaele University, 20132 Milan, Italy; 2 Institute of Psychology, Jagiellonian University, 31-007 Krakow, Poland

**Keywords:** dating apps, prejudice, social perception, social media, online dating, romantic relationships, Tinder

## Abstract

While dating apps such as Tinder are now widespread, social stigma toward their users remains a significant yet underexplored issue. Two experiments examined how prejudice toward Tinder shapes social perception and interpersonal attraction. Study 1 (*N* = 206) investigated impressions toward a potential partner whose profile was described as originating from Tinder versus Facebook. Higher prejudice predicted reduced attention to the target’s images when the profile was presented as coming from Tinder and was associated with slightly more negative trait attributions. Curiously, participants with experience using dating apps appeared to be less attracted to the Tinder user. Study 2 (*N* = 481) tested evaluations of couples formed offline, on Facebook, or on Tinder. Online-formed couples—regardless of platform—were judged more negatively than offline couples. Furthermore, a form of projected embarrassment emerged, whereby participants believed that Tinder-initiated couples, more than others, would be less willing to tell others about their true origin. Together, these findings document a persistent bias toward online dating. The implications of these results are discussed in relation to the striking paradox between this enduring social stigma and the documented success of some online-formed bonds, highlighting how such prejudice may ultimately preclude individuals from accessing significant relational opportunities.

## 1. Introduction

Today, the increasing digitalization of intimate social interactions has made dating applications (DAs), particularly Tinder, a dominant way for initiating romantic relationships (Fisher & Garcia, 2019; Rosenfeld et al., 2019; Ranzini & Lutz, 2017). Despite their ubiquity, however, dating apps are often framed negatively by both non-users and users, leading to a pronounced social stigma towards Tinder users and/or online-formed couples (Anderson, 2005; Donn & Sherman, 2002; Sprecher et al., 2008; Wildermuth, 2004). Although this stigma appears to be less rooted in the stereotypes that online dating users are dangerous or deviant individuals (Finkel et al., 2012), it still links DAs primarily to superficiality, occasional encounters, and low self-esteem (Sumter et al., 2017; Strubel & Petrie, 2017; Holland & Tiggemann, 2016). Nevertheless, the social perceptions underlying this stigma remain significantly underexplored.

### 1.1. Stereotyping and Devaluating the Typical User: The “Tinder Stamp”

In social life, prejudice functions as a cognitive schema that pre-determines negative attitudes toward individuals belonging to a certain social group. When applied to digital contexts and online dating, these schemas may trigger a process of generalization where the perceived technological affordances of Tinder—such as swiping based on physical appearance or choosing a potential partner from a digital showcase—are reflexively attributed to its Tinder users. This process often results in a negative halo effect, where Tinder use, for instance, may be interpreted as a preference for occasional encounters based on sexual/physical attraction (Krüger & Spilde, 2020). Additionally, according to the social compensation hypothesis (Valkenburg & Peter, 2007), observers may assume that DAs users are unable to find a partner through traditional means, perceiving them as socially unsuccessful or “lame”.

Although research on prejudice toward DAs remains scarce, initial empirical evidence supporting and exploring the content of such prejudice comes from recent experimental research. In a series of five studies, Silva et al. (2019) investigated how people perceive Tinder users, specifically focusing on perceived trustworthiness. They hypothesized that Tinder users are subject to a systematic devaluation compared to users of other platforms (e.g., Facebook) due to the prevailing narrative identifying Tinder as a “hook-up app”, primarily used for casual encounters. Their results confirmed that profiles associated with Tinder were judged as significantly less trustworthy than identical profiles presented on platforms perceived as being more relationship-oriented or within general social contexts. Interestingly, the authors found support for the persistence of what they called a “Tinder stamp”: the devaluation was not limited to the immediate context of the app—as long as the targets were presented as utilizing the app—but endured even when individuals were later evaluated in neutral contexts where the original platform association had been removed. Additionally, Tinder users were perceived as less trustworthy than users of more “serious” dating sites—specifically those that do not rely on the algorithmic swiping mechanic.

In another quasi-experimental work, Johanis et al. (2024) explored the social stigma associated with online dating, comparing users and non-users’ perceptions. They reported that individuals seeking partners via digital platforms are generally perceived as more desperate and less socially desirable than those utilizing traditional offline methods. Specifically, the negative stereotypes associated with DAs portrayed the typical user as a superficial individual, primarily driven by transient sexual motives or lacking the essential social skills required to succeed in traditional meeting contexts. Crucially, the study identified a significant discrepancy in perceptions based on the evaluator’s personal experience with these platforms: While non-users showed robust negative biases—projecting a negative halo of social inadequacy onto app users—current users tended to normalize the practice, viewing it more as a rational and strategic choice. These findings suggest that familiarity with the platform—having used it in the past—may act as a buffer, shifting the perception of online dating from a sign of social ineptitude to a legitimate and normalized tool for modern socialization.

Preliminary findings regarding the content of this prejudice also emerge from qualitative research. Through a series of interviews with Tinder users, Degen and Kleeberg-Niepage (2022) found that the platform is frequently framed as a “hook-up app” or a “blunt game”, with users often speaking pejoratively about the profiles found there and labeling other users as “desperate”. Interestingly, while users may claim that online dating is now normalized (Johanis et al., 2024), they simultaneously tend to express disdain for the platform. This suggests a form of normalized prejudice, where users tend to distance themselves from the “typical” Tinder user (perceived as superficial or desperate) even while actively using the platform. In doing so, they attempt to counteract the stigma and maintain a positive social identity despite negative stereotypes (see the phenomenon of social distancing from one’s own in-group; Tajfel & Turner, 1979; see van Veelen et al., 2020, for a review).

Similarly, the results of Degen and Kleeberg-Niepage (2022) also show that prejudice exists among Tinder users themselves, in a clear sign of self-stereotyping (Biernat et al., 1996; see also Latrofa et al., 2010). Through the lens of the negative halo effect, profile features are often interpreted as confirming stereotypes: for instance, highly curated photographs may be viewed as signs of vanity or an attempt to manipulate the viewer’s impressions, rather than standard self-presentation (e.g., Hancock & Toma, 2009; Parsa et al., 2022; Fang & Lee, 2023). Likewise, the absence of a linked Instagram account may foster the impression that the user has something to hide and/or lacks a sufficiently “popular”, active social life, reinforcing the desperate stereotype (e.g., Duguay, 2017). In short, the mere association with the platform functions as a negative filtering cue that tends to overshadow users’ positive traits and characteristics while simultaneously attributing to them the negative ones typically ascribed to Tinder users, even on the platform and among users themselves.

### 1.2. Beyond the Individual: The “Stigma of Origin” Toward Online-Formed Relationships

This devaluation extends beyond the individual level, also affecting how romantic relationships are perceived. Online-formed couples seem to face a “stigma of origin”, where their bond is often viewed through the lens of relational fragility (Wildermuth, 2004; Riger, 2017). For instance, when a couple’s meeting is attributed to an algorithm or a “low effort” digital encounter, external observers tend to perceive the relationship as lacking the organic foundation or destiny associated with offline meetings, ultimately judging the bond as less serious or less likely to persist over time. Incredibly, negative prejudice appears to persist even toward well-established relationships (Riger, 2017). Regardless of the couple’s longevity, the online origin may potentially lead to negative interpretations of the partners’ motives, consistently biasing the overall evaluation of the bond that condition the evaluation of the couple.

The reality of such a devaluation by others is reflected in how users themselves manage their social image. In her qualitative analysis, Riger (2017) investigated how users of dating applications shape and share the narratives of their online encounters with others. The author documented that individuals who met their partners online often edit or hide the origins of their stories, sharing them honestly and completely only with trusted audiences as a strategy to anticipate and prevent negative judgment.

Nevertheless, a growing body of research suggests that, while the stigma toward DAs persists, users increasingly recognize that the practical benefits of these platforms outweigh the social costs. Empirical evidence confirms that Tinder encounters, when moved offline, often lead to committed relationships and marriage, rather than just casual hook-ups (e.g., Machfudz et al., 2021; Sharabi, 2023). For instance, Timmermans and Courtois (2018) found that while some Tinder matches led to casual sex, more than a quarter resulted in the formation of a committed relationship. These findings, according to the authors, challenge the “hookup app” stereotype. Indeed, sexual encounters might increasingly serve as a precursor to committed relationships in modern society.

In sum, research reveals an apparent stigma attached to relationships that originate from Tinder. More generally, couples whose initial interaction occurred online seem to be frequently perceived as more casual, less trustworthy, and less legitimate than those with offline beginnings, even though the lived experiences and long-term outcomes of these couples appear to be largely similar. As highlighted by Potarca (2020), there are no significant differences between couples initiated through dating apps and those initiated elsewhere in terms of relationship and life satisfaction.

### 1.3. The Present Research

While research has extensively focused on the motives for using dating apps like Tinder and the psychological impact of such use (e.g., Gatter & Hodkinson, 2016; Holland & Tiggemann, 2016), experimental evidence on how external observers’ prejudice influences their perceptions and evaluations remains scarce. We propose that high-prejudice observers evaluate both Tinder users and the relationships they form more negatively. The intent of this work was not only to better explore the content of a pervasive social stigma but also to document the persistence of this stigma beyond the initial encounter, showing how even successful, long-term bonds can be “tainted” by their digital origin. Furthermore, given the current lack of research grounded in theoretical frameworks addressing the social perception of dating app users and online-formed couples, we hope this research can stimulate theorization on the psychosocial processes underlying this stigma.

In the Italian context, where the present research was conducted, Tinder serves as a prototypical representative of dating apps, frequently used in common parlance as a shorthand for the entire category of digitally mediated encounters and widely recognized by non-users (e.g., YouGov, 2021). As Tinder is especially popular among young adults (e.g., Sawyer et al., 2018; Iovino, 2026)—the primary demographic in our convenience samples—we employed it as our primary focal point. Conversely, we used Facebook (Esposito, 2024) as a contrast platform to represent digitally mediated but socially driven encounters, as opposed to intentional, app-based dating.

The research included two experiments designed to investigate how prejudice toward Tinder shapes social perception at two distinct but complementary levels: the individual and the relational. In Study 1, we focused on the evaluation of a potential partner met online, predicting that the platform source (Tinder vs. Facebook) would interact with participants’ pre-existing prejudice in determining their impressions of the targets. Specifically, we hypothesized that higher prejudice would function as a negative filter, leading participants to allocate less attention to the target’s images, experience less attraction toward that potential partner, and attribute fewer positive (and more negative) traits to them when presented as a Tinder user compared to a Facebook user.

Building on these individual-level findings, Study 2 examined whether this stigma generalizes to the relational level, comparing couples formed offline, on Facebook or on Tinder. Consistent with the bias predicted in Study 1, we hypothesized that a negative prejudice would emerge against couples formed through any online platform—whether Facebook or Tinder—leading them to be judged less favorably in terms of quality and stability compared to those formed offline. However, beyond this general “online stigma”, we also expected a specific, incremental prejudice toward DAs: couples originating from a Tinder match were predicted to be attributed the fewest positive and the most negative characteristics among all groups, reflecting a specific bias against dating app environments. The present research was reviewed and approved by the Institutional Review Board (IRB) of the Jagiellonian University.

## 2. Study 1

### 2.1. Materials and Methods

#### 2.1.1. Participants, Design, and Sensitivity Analysis

A total of 206 young adults residing in Italy (44.7% female; *M*_age_ = 22.15, *SD* = 3.32) volunteered in the experiment. Inclusion criteria required participants to be currently single and to have never participated in psychology-related studies before. As many participants were students at institutions where psychology studies are frequently conducted, this latter criterion was employed to minimize participant sophistication and ensure that all respondents were naive to experimental manipulations and social psychology paradigms. Regarding gender attraction and sexual orientation, 53.4% reported being attracted to women, 43.7% to men, and 2.9% to both. The majority of the sample reported being heterosexual (90.8%), while 6.3% identified as homosexual (7 males attracted to men and 6 females attracted to women) and 2.9% as bisexual (attracted to both; *n* = 6). The study employed a 2 (Prejudice Level: Low vs. High) × 2 (Profile Source: Facebook vs. Tinder) between-subjects design. The dependent variables were time spent on the target profile, perceived attraction, and trait attribution (positive and negative personality characteristics). The study had 80% power to detect an effect size of at least *f* = .20 in main and interaction effects (*α* = .05; groups: 4; numerator *df*: 1; non-centrality parameter *λ* = 7.92; G*Power 3.1, Faul et al., 2007).

#### 2.1.2. Procedure

Participants were recruited online through recruitment announcements on various social media platforms (e.g., Facebook, Instagram, and WhatsApp), targeting, for example, university student groups. The study was framed as an investigation into the psychological dynamics of romantic relationship formation online. After providing informed consent and receiving instructions to ensure a distraction-free environment, participants completed a three-part survey administered online via Qualtrics.

The first section collected demographic data and social media frequency of use (i.e., “In general, how frequently do you use social networking sites?”), including information regarding participants experience with dating apps (e.g., “Have you ever used a dating app in the past?”; “Do you currently have a profile on any dating app?”). It also assessed prejudice toward Tinder to categorize participants into high or low prejudice groups. In the second section, participants were shown a social media profile of a potential partner (either a male or female target, matched to the participant’s sexual orientation; those attracted to both sexes were randomly assigned to one of the two targets). To manipulate the profile source, participants were randomly assigned to either a Facebook (control) or Tinder (experimental) condition. Finally, the third section measured the dependent variables: dedicated attention (dwell time), interpersonal attraction, and the attribution of positive (e.g., kindness) and negative (e.g., superficiality) personality traits.

All respondents volunteered for the study without incentives, and they were informed that their contribution would help advance scientific knowledge regarding social perception in online dating contexts. At the end of the procedure, participants were fully debriefed and thanked for their precious participation.

#### 2.1.3. Materials

**Background Measure of Prejudice Toward Tinder.** To assess participants’ prejudice toward Tinder, we first provided a brief description of the application to ensure that even those unfamiliar with it could respond. Prejudice was then measured using a 10-item scale ad hoc developed for this study (e.g., “People who use Tinder are insecure and unable to find partners in real life”; “It is impossible to meet serious people through Tinder”; “People on Tinder do not show who they truly are”; “People who use Tinder are desperate”). Responses were recorded on a Likert-like scale ranging from 1 (*absolutely false*) to 5 (*absolutely true*). The mean of these ten items constituted the participant’s prejudice score, with higher scores indicating higher levels of prejudice (Cronbach’s *α* = 0.83). To later test our hypotheses, a median split (*Mdn* = 2.80) was performed on the total scores to split participants into two groups: low prejudice (*n* = 107; *M* = 2.29; *SD* = 0.40) and high prejudice (*n* = 99; *M* = 3.32; *SD* = 0.34).

**Figure 1:**
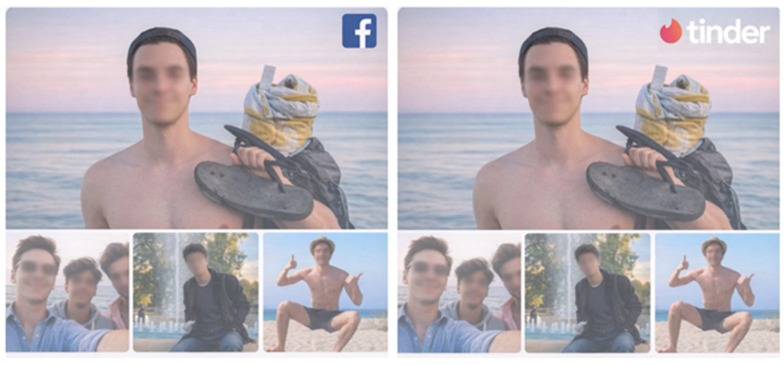
Examples of the Male Target’s Photos for the Facebook and Tinder Conditions (Study 1).

**Manipulation of the Target Profile Source.** To evaluate the effects of prejudice toward Tinder on the perception of a potential partner, participants were randomly assigned to one of two experimental conditions. Participants were asked to evaluate the online profile of a potential partner. In the control condition, participants were told that the target’s profile originated from Facebook, while in the experimental condition participants believed that the same profile originated from Tinder. Importantly, to later ensure a clearer interpretation of our findings, in both conditions, we simply stated that the target’s photos were sourced from Facebook or Tinder, without providing further details that could frame the platform—especially in the case of Facebook—in a romantic or “dating” key. Crucially, the visual and textual stimuli remained identical across conditions: all participants viewed the same set of six photographs—of a male or female target, depending on their orientation—and the same biographical description. The only variation concerned the branding, which identified the source as either Facebook or Tinder (see Figure 1). The full text of the experimental instructions is provided in the Supplementary Materials (Section S1).

The target profiles consisted of six high-resolution photographs depicting the target in both individual and social settings. To ensure consistency, the male and female target profiles were matched for attractiveness and followed a similar photographic structure (both presented two close-up portraits, two medium-range shots, and two photographs with other people). The profile’s biography was also identical for male and female targets; the full biographical text is available in the Supplementary Materials (Section S1).

**Dependent Measures: Time Spent on the Target’s Profile, Attraction, and Attribution of Positive and Negative Traits.** The dependent measures of the study were the following: time spent viewing the target’s social profile, attraction toward the target, and the attribution of positive and negative personality traits to the target. Qualtrics recorded the dwell time for each participant, measuring the duration of exposure to the target’s photos before they proceeded to the subsequent section of the survey. Attraction and personality trait attributions were assessed through self-report questions. All dependent measures were administered only after participants had viewed the target’s photographs and the brief biographical statement.

Attraction toward the target was measured using four items: “Overall, how attracted do you feel to Marco/Alice?”, “Do you find Marco/Alice physically attractive?”, “Do you find this person interesting?”, and “How much do you think you would like to go on a date with Marco/Alice?”. Responses were provided on an 11-point Likert scale ranging from 0 (*not at all*) to 10 (*very much*). The four items showed high internal consistency (*α* = 0.92) and were averaged to create a composite attraction score.

Participants’ expectations regarding the target’s personality traits were assessed with the following prompt: “If you had to imagine Marco’s/Alice’s personality, how would you expect it to be?”. Participants were then presented with a series of positive traits—interested in a serious relationship, kind and affectionate, sensitive to my needs, sociable, intelligent, and humorous (5 items; *α* = 0.60)—as well as a series of negative traits—jealous, dishonest, distant, superficial, and unfaithful (5 items; *α* = 0.77). For each trait, participants indicated their level of agreement using a 5-point Likert scale ranging from *absolutely not* to *absolutely yes*.

#### 2.1.4. Data Analysis Note

The analytical strategy of treating prejudice with a median split was conceptually driven. Although prejudice was measured as a continuous variable, we primarily adopted a group-based approach using a median split (high vs. low prejudice) to identify distinct psychological profiles and facilitate the interpretation of interactions—a standard approach in experimental social psychology when the aim is to identify the boundary conditions of an effect. Nonetheless, the use of median splits is a frequent and established practice precisely in those studies where prejudice is expected to moderate the effects of other manipulated variables (e.g., Martinovic & Verkuyten, 2013; von Hippel et al., 1997; West et al., 2017). To complement our findings and ensure the robustness of our results, we also performed supplementary linear regressions treating prejudice as a continuous variable.

### 2.2. Results

#### 2.2.1. Preliminary Analyses

Before testing our main hypotheses, we conducted descriptive analyses and bivariate correlations among all dependent variables. Descriptive statistics for all the main dependent variables of the present study are summarized in Table 1. Attraction was positively correlated with the attribution of positive traits, *r*(204) = 0.32, *p* = 0.001, and negatively correlated with negative traits, *r*(204) = −0.15, *p* = 0.027. Positive traits attribution negatively correlated with negative traits attribution, *r*(204) = −0.38, *p* = 0.001. Planned preliminary checks confirmed that the randomization was successful, as no significant differences in demographic variables (age, gender) were found across the experimental conditions, with the groups showing similar age, *t*(204) = 0.90, *p* = 0.367, and similar gender distributions, *χ*^2^(1, *N* = 206) = 0.43, *p* = 0.512. Finally, preliminary analyses also indicated no significant differences in prejudice levels based on participants’ sexual orientation—heterosexual (*n* = 187), homosexual (*n* = 13), or bisexual (*n* = 6), *F*(2, 203) = 1.06, *p* = 0.347.

Regarding the two different evaluation targets implemented in the present procedure (male or female), a planned statistical comparison revealed that the female target was rated as significantly more attractive than the male target, *F*(1, 204) = 19.99, *p* = 0.001. The female target also received slightly higher ratings for the attribution of negative traits, *F*(1, 204) = 3.96, *p* = 0.048. Importantly, target gender did not significantly interact with any of the factors included in the main research design (i.e., prejudice, profile source, or their interaction). Given these preliminary checks, target gender was not further distinguished in the following reports.

Before testing our main hypotheses, to provide a descriptive overview, we assessed participants’ familiarity with online dating and social media. Familiarity with Tinder was almost universal (98.5%), with a third of the sample (34%) reporting they knew the platform through past or current use. Regarding social media engagement, the vast majority of the sample reported regular use—68% used social media “often” or “very often”—while only a small minority (0.5%) reported having no social media presence. Additionally, participants reported using social media platforms to nurture existing romantic relationships (20%), with 14.1% of the sample indicating they “often” or “very often” use these sites to initiate new romantic interests or seek a relationship.

Regarding specific experience with dating apps, 35% of participants declared they had previously created a profile on at least one app, while a smaller portion (26.2%) reported they were currently maintaining an active profile. Furthermore, a consistent part of the sample expressed an interest in continuing to use or trying a dating app for the first time in the future (totaling 59.2%; “maybe”: 32.5%; “probably yes”: 22.8%; “absolutely yes”: 3.9%).

#### 2.2.2. Dedicated Attention: The Interaction Between Prejudice and Profile Source

**Figure 2:**
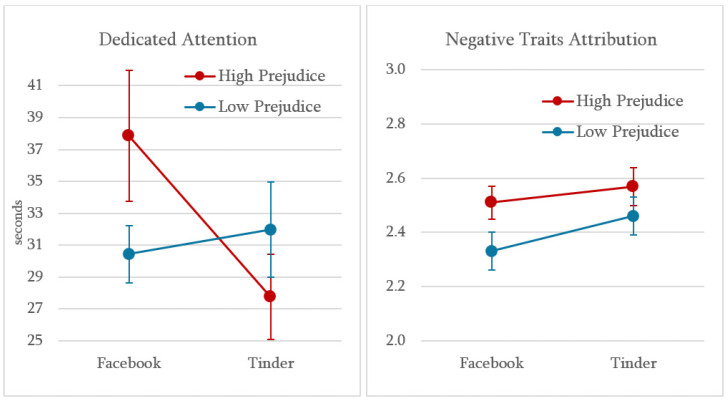
The Effects of Prejudice Toward Tinder and Profile Source on Attention Dedicated to a Potential Partner’s Profile and the Attribution of Negative Traits (Study 1). *Note.* The graphs show the average number of seconds spent viewing the target profile (**left panel**) and the scoring of attribution of negative traits to the target (**right panel**) as a function of pre-existing prejudice toward Tinder (low vs. high) and the profile’s manipulated source (Facebook vs. Tinder). Error bars represent standard errors.

We first examined whether prejudice predicted the amount of time participants spent viewing the target’s profile. In line with our hypotheses, a 2 (Prejudice: low vs. high) × 2 (Profile Source: Facebook vs. Tinder) between-subjects ANOVA on dwell time revealed a significant interaction between prejudice and manipulated profile source, *F*(1, 202) = 3.95, *p* = .048, *η_p_*^2^ = .019 (Figure 2). Simple effects analysis indicated that high-prejudice participants spent significantly less time viewing the profile when it was presented as coming from Tinder compared to when it was presented as coming from Facebook, *t*(78.91) = 2.06, *p* = .043 (see Table 1). For low-prejudice participants, no such difference in dwell time was observed across conditions (Tinder: *M* = 31.96; *SD* = 21.66; Facebook: *M* = 30.44; *SD* = 13.18), *t*(205) = 0.44, *p* = .660. This reduction in dwell time may potentially reflect the application of a cognitive schema. As prejudice functions as a cognitive shortcut, it is possible that high-prejudice individuals rely on pre-existing stereotypes to quickly form an impression of the Tinder user, thereby requiring less time to process the potential partner’s information. The main effects of prejudice, *F*(1, 202) = 0.30, *p* = .585, *η_p_*^2^ = .001, and profile source, *F*(1, 202) = 2.15, *p* = .144, *η_p_*^2^ = .011, were both non-significant. A supplementary linear regression confirmed this pattern: although the interaction term did not reach conventional significance, *p* = .173, a simple slope analysis showed that at high levels of prejudice (mean +1 SD), the difference between the Facebook and Tinder conditions approached significance, *p* = .054.

#### 2.2.3. The Absence of Effects on Interpersonal Attraction

Regarding interpersonal attraction, the 2 × 2 ANOVA yielded no significant main effects for either prejudice toward Tinder, *F*(1, 202) = 0.52, *p* = .473, *η_p_*^2^ = .003, or profile source, *F*(1, 202) = 0.88, *p* = .350, *η_p_*^2^ = .004. The interaction effect was also non-significant, *F*(1, 202) = 1.19, *p* = .277, *η_p_*^2^ = .006 (Table 1). These results indicate that neither the profile platform’s label nor participants’ prior prejudice directly influenced the overall attraction toward the target. A supplementary regression analysis treating prejudice as a continuous variable further confirmed this lack of significant main or interaction effects, *p*_s_ ≥ .230, consistently showing that interpersonal attraction was not affected by the experimental manipulation and the measure of prejudice, regardless of the analytical approach.

#### 2.2.4. The Effects of Prejudice on Personality Trait Attribution

To test the hypothesized impact of prejudice and profile source on the perception of the target personality, two 2 × 2 between-subjects ANOVAs were performed on positive and negative traits attribution (Table 1). Regarding positive traits, the ANOVA yielded no significant main or interaction effects, *F_s_*(1, 202) ≤ 0.30, *p_s_* ≥ .585, *η_p_*^2^*_s_* ≥ .001, suggesting that participants’ expectations of desirable qualities were not influenced by the profile source or prior beliefs about Tinder.

As to the attribution of negative traits, results revealed a significant main effect of prejudice, *F*(1, 202) = 4.16, *p* = .043, *η_p_*^2^ = .020 (Figure 2). As hypothesized, participants in the high-prejudice group attributed slightly but significantly more negative traits to the target (*M* = 2.54; *SD* = 0.49) compared to those in the low-prejudice group (*M* = 2.40; *SD* = 0.52), regardless of the target profile’s source. No significant main effect of profile source, *F*(1, 202) = 1.99, *p* = .160, *η_p_*^2^ = .010, or interaction effect, *F*(1, 202) = 0.24, *p* = .623, *η_p_*^2^ = .001, was found for negative traits (low prejudice, Tinder: *M* = 2.46; *SD* = 0.48; low prejudice, Facebook: *M* = 2.33; *SD* = 0.55; high prejudice, Tinder: *M* = 2.57; *SD* = 0.54; high prejudice, Facebook: *M* = 2.51; *SD* = 0.44).

For both positive and negative trait attributions, supplementary continuous regression models did not yield significant main or interaction effects, *p*_s_ ≥ .129. This might suggest that these perceptual differences are primarily observable when comparing distinct prejudice-based groups rather than across a continuous spectrum. While these regression results did not reach conventional significance levels and must therefore be interpreted with caution, the directional trends remained consistent with our primary findings.

#### 2.2.5. Additional Analyses: Attraction and the Paradox of Past Experience with Dating Apps

Given that the primary models did not always yield the expected results with respect to interpersonal attraction and trait attribution, we conducted exploratory analyses to examine whether participants past experience with dating apps (DAs) was somehow associated with their evaluations. As might be expected, we first observed an association between experience and prejudice: participants who had used a dating app at least once in the past (*n* = 72) reported lower levels of prejudice (*M* = 2.53; *SD* = 0.65) compared to non-users (*n* = 133; *M* = 2.92; *SD* = 0.59), *t*(203) = 4.28, *p* < .001. Similarly, a significant negative correlation emerged between participants’ preexisting prejudice toward Tinder and their interest in using (or continuing to use) a dating app in the future, *r*(202) = −0.33, *p* < .001, suggesting that higher prejudice was associated with lower future intention.

**Figure 3:**
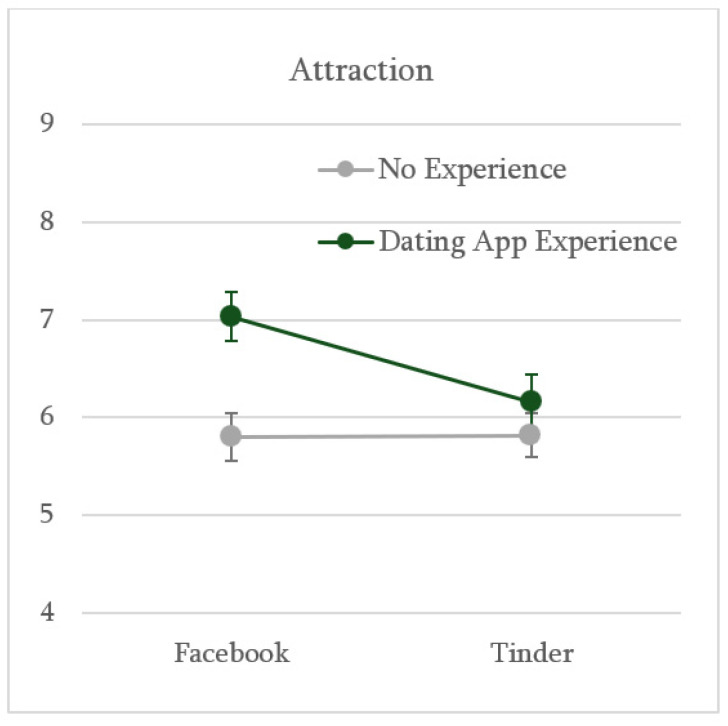
The Effects of Past Experience with a Dating App and Target Profile Source on Attraction (Study 1). *Note.* The graph shows the average levels of reported attraction toward the target as a function of past experience with a dating app (no vs. yes) and the manipulated source of the target’s profile (Facebook vs. Tinder). Error bars represent standard errors.

In contrast, including past experience with a dating app as a factor in our models led to less predictable and more nuanced results. Specifically, a 2 (Profile Source: Facebook vs. Tinder) × 2 (Past Experience: no vs. yes) ANOVA revealed two interesting findings (Figure 3). First, we found a main effect of past experience: participants with experience evaluated the target as generally more attractive (*M* = 6.55; *SD* = 1.66) than those without experience (*M* = 5.81; *SD* = 1.86), *F*(1, 201) = 8.94, *p* = .003, *η_p_*^2^ = .043.

Second, and most paradoxically, the exploratory analysis revealed a marginally significant interaction effect between manipulated profile source and past experience on reported attraction, *F*(1, 201) = 2.94, *p* = .088, *η_p_*^2^ = .014 (Figure 3). Simple effects analysis showed that participants with past DA experience reported significantly lower attraction toward the target when the profile was presented as coming from Tinder (*n* = 40; *M* = 6.16; *SD* = 1.76) compared to when it was presented as coming from Facebook (*n* = 32; *M* = 7.03; *SD* = 1.40), *t*(70) = 2.29, *p* = .025. Conversely, for participants without past experience, the profile source did not significantly affect attraction, which remained relatively and independently low across both conditions (Facebook: *n* = 67; *M* = 5.80; *SD* = 1.95; Tinder: *n* = 66; *M* = 5.82; *SD* = 1.79), *t*(131) = 0.07, *p* = .942. Notably, participants’ gender did not interact with any of these factors in influencing attraction, *F*_s_(1, 197) ≤ 1.70, *p*_s_ ≥ .193.

### 2.3. Discussion

Taken together, the findings of Study 1 are different than expected. The observed main effect of prejudice—where negative trait attribution increased regardless of the platform (Tinder vs. Facebook)—suggests that a prior prejudice toward Tinder may have led to a more negative perception of *any* potential partner presented in an online setting, whether the profile is encountered on Facebook or on Tinder itself. In this sense, the stigma associated with Tinder might have generalized to the broader digital dating context, leading high-prejudice individuals to evaluate any online potential partner through a more critical lens. However, as our design lacked an offline control condition, this remains a tentative, exploratory explanation for why Study 1 did not yield the predicted platform-specific effects. Notably, this bias appears to be selectively associated with an increased attribution of negative characteristics, without being associated with the perception of positive traits.

Regarding past experience, the finding that users with DAs experience evaluated both targets as generally more attractive suggests a familiarity effect. First-person experience with dating apps may lead individuals to evaluate online profiles more favorably, even when the profile is not explicitly hosted on a dating platform but is simply presented within a “potential partner” frame. In other words, when experienced users are tasked with evaluating another user in romantic terms, they appear to be more appreciative—or perhaps less judgmental—than their non-experienced counterparts.

If supported by further empirical evidence, this interpretation could clarify the previously observed pejorative effect of prejudice on the attribution of negative traits—which unexpectedly appeared generalized across both Facebook and Tinder and sounded somewhat inexplicable at first. It is possible that simply presenting a target as a “potential partner” in a digital setting is sufficient to activate certain stereotypes, regardless of the specific platform. Future research should consider comparing the presentation of a target within a romantic/dating frame versus a neutral frame (e.g., “What traits would you attribute to this potential partner?” vs. “this person?”) to further isolate this effect.

The marginally significant interaction effect between manipulated profile source and past experience on reported attraction also has interesting potential explanations. This paradoxical finding may suggest that even “insiders” may harbor a latent prejudice or experience a sort of “dating app fatigue” that perhaps biases their evaluation of potential partners specifically when encountered within the Tinder environment.

## 3. Study 2

### 3.1. Materials and Methods

#### 3.1.1. Participants, Design, and Sensitivity Analysis

A total of 481 young adults residing in Italy (68.8% female; *M*_age_ = 21.17, *SD* = 3.25) volunteered in the experiment. Participants were recruited regardless of their current relationship status; however, as in Study 1, we exclusively recruited individuals with no prior experience in psychology studies to minimize participant sophistication and ensure they remained naive to the experimental manipulations. The majority declared they were single (79.4%), while the others were either in a stable relationship (7.7%) or in the early stages of one (12.7%). The study implemented a one-factor (Origin Condition: offline vs. Facebook vs. Tinder) between-subjects design, where the attribution of positive and negative relationship characteristics to a target couple served as the dependent variables. A sensitivity analysis indicated that the study had 80% power to detect an effect size of at least *f* = 0.14, that is a small-to-medium effect (*α* = .05; groups: 3; numerator *df*: 2; non-centrality parameter *λ* = 9.70; G*Power 3.1, Faul et al., 2007).

#### 3.1.2. Procedure

Participants were recruited through a mixed-sampling approach, using both online social media platforms (e.g., Facebook, Instagram, and WhatsApp)—targeting primarily university student groups—and offline snowball sampling. The study was framed as an investigation into social perceptions of established romantic relationships. After providing informed consent, participants completed a three-part web-based survey administered on Google Forms. Mirroring the structure of Study 1, the first section collected demographic data and social media usage frequency, alongside assessments of participants’ familiarity with Tinder and their broader experience with dating apps and social media for romantic purposes (e.g., “Do you know Tinder?”; “Have you ever used a dating app in the past?”). In the second section, participants were presented with a standardized narrative describing a young romantic couple, accompanied by three photographs depicting the partners together in everyday settings. To manipulate the relationship origin, participants were randomly assigned to one of three conditions that varied only the context of the couple’s first meeting: offline, Facebook, or Tinder. Finally, the third section assessed the dependent variables through a series of items requiring participants to attribute, according to their first impression, specific relational characteristics to the couple, resulting in scores for both positive and negative characterization. Participants volunteered without financial or academic incentives; at the end of the study, they were fully debriefed and thanked after being informed that their contribution would advance scientific knowledge on social perception and prejudice toward online dating.

#### 3.1.3. Materials

**Manipulation of the Relationship Origin.** Participants were randomly assigned to one of three experimental conditions: offline (control), Facebook, or Tinder. All participants read a standardized description of a young couple’s relationship history. In the offline condition, the partners were described as having met at a bar; in the two online conditions, the meeting was portrayed as having taken place on Facebook or a dating app (i.e., Tinder). For the complete prompt and the version used in each condition, see Supplementary Materials (Section S2).

To help participants form a more complete and realistic impression of the targets, the description was accompanied by three photographs of the couple. After carefully reading the narrative and observing the images, participants proceeded with the questionnaire entailing the measures for the dependent variables. Keeping all biographical details and visual stimuli identical across conditions ensured that any observed differences in the attribution of relationship characteristics could be uniquely attributed to the meeting context.

**Dependent Measures: Positive and Negative Characterization of the Evaluated Couple.** To assess participants’ perceptions of the couple following the experimental manipulation, a 22-item questionnaire was administered. These items required participants to evaluate both the partners and the nature of their relationship. The items were specifically developed for this research to capture the nuanced social stigmas and expectations associated with the meeting contexts examined (e.g., the “superficiality” or “instability” often attributed to online dating). To minimize social desirability bias, the phrasing was carefully curated to avoid evaluative language; for instance, judgmental terms such as “to judge” were strictly avoided in the questionnaire. The instrument included two distinct subscales: positive and negative characterization. The positive characterization scale consisted of 15 items assessing positive relational traits and characteristics, such as emotional bond and harmony (e.g., “According to your impression, do the two partners have real feelings for each other?”; “Are the two partners bound by deep intimacy and connection?”; “Are they sincere with each other?”; Cronbach’s *α* = .83). The negative characterization scale included 7 items capturing negative impressions as well as signs of potential relational instability (e.g., “Could the two partners betray each other in the future?”; “Is their bond based solely on physical attraction?”; “Are they settling for one another?”; Cronbach’s *α* = .75). For all items, participants recorded their impressions on a 9-point Likert-like scale ranging from 1 (*extremely unlikely*) to 9 (*extremely likely*). In both the scales, higher scores reflected a higher attribution of those characteristics.

### 3.2. Results

#### 3.2.1. Preliminary Analyses

Before proceeding with hypothesis testing, we conducted descriptive analyses and calculated a bivariate correlation among the two dependent variables. Table 2 summarizes the descriptive statistics for each experimental condition. A weak negative correlation was found between levels of positive and negative characterization, *r*(479) = −0.17, *p* < .001. Preliminary checks also confirmed that no significant differences in demographic variables (age, gender) emerged across the experimental conditions, with the groups showing similar age, *F*(2, 478) = 0.25, *p* = .779, and gender distributions, *χ*^2^(4, *N* = 481) = 4.18, *p* = .383.

Regarding participants’ familiarity with online dating and social media, results revealed that nearly all participants were already familiar with Tinder at the time of the study (96.9%). The majority of the sample reported using social media platforms “often” (40.1%) or “very often” (23.7%), while only a negligible minority (0.2%) reported never using them. Furthermore, most participants indicated they did not typically use social media platforms to nurture existing romantic relationships (69.4%). Conversely, 15.8% of the sample reported that they “often” or “very often” used these sites to initiate new romantic interests or seek a relationship.

Regarding specific experience with dating apps, 32.6% of participants declared they had already used them at least once in the past, while 19.5% reported they were currently maintaining an active profile. Mirroring what we observed in Study 1, a substantial portion of the sample expressed an interest in either continuing to use or trying a dating app for the first time in the future (53.6%), with participants indicating they would “maybe” (32.6%), “probably” (16.6%), or “absolutely” (4.4%) consider using these platforms again or for the first time.

#### 3.2.2. The Absence of Effects of Origin Condition on the Positive Characterization of the Couple

To test our first hypothesis, a one-way ANOVA was conducted to examine the effect of the relationship origin condition (offline vs. Facebook vs. Tinder) on the positive characterization of the evaluated couple. The analysis yielded no significant effect, *F*(2, 478) = 0.87, *p* = .419, *η_p_*^2^ = .004. This result indicates that the context of the couple’s first meeting did not directly influence participants’ attribution of positive relational traits (e.g., those inherent to their emotional bond, harmony, or other relationship qualities). Regardless of whether the partners were described as having met at a bar, on Facebook, or through Tinder, their relationship was perceived as having comparable levels of positive characteristics (Table 2).

Exploratory item-level analyses revealed a noteworthy result. Among those assessing positive characterization, one single item was particularly sensitive to the manipulation of the relationship’s origin: participants were asked whether they believed the partners would openly tell others how they met. A clear effect of the manipulated relationship origin emerged, *F*(2, 478) = 22.15, *p* < .001, *η_p_*^2^ = .085, such that couples who met online—and especially those who met on Tinder—were perceived as less willing to disclose the circumstances of their meeting (offline: *M* = 6.91; *SD* = 1.82; Facebook: *M* = 5.85; *SD* = 2.35; Tinder: *M* = 5.38; *SD* = 2.28). Specifically, LSD-corrected pairwise comparisons revealed that online origin conditions were consistently evaluated as less willing to disclose their origin than the offline condition, *t*_s_ ≥ 4.54, *p*_s_ < .001. Furthermore, a marginally significant difference emerged between the two online conditions, with the couple that started dating on Tinder being judged as less likely to share their origin than the Facebook couple, *t*(306) = 1.78, *p* = .056.

#### 3.2.3. The Effects of Relationship Origin on Negative Characterization: Unpacking the Nature of Prejudice Toward Online-Formed Couples

In contrast, a one-way ANOVA revealed a significant and robust main effect of the origin condition on the negative characterization of the couple, *F*(2, 478) = 12.97, *p* < .001, *η_p_*^2^ = .051. LSD-corrected pairwise comparisons showed that couples whose relationship originated online—whether on Facebook or Tinder—were attributed significantly higher levels of negative characteristics compared to those who met offline, *t*_s_ ≥ 3.59, *p*_s_ < .001 (Table 2). Importantly, we did not find a significant difference between the Facebook and Tinder conditions, with the Facebook-origin couple receiving similar negative evaluations to the Tinder one, *t*(306) = 1.20, *p* = .230.

To further investigate the specific nature of this negative characterization, we conducted exploratory item-level analyses to identify which relational aspects were most affected by the origin experimental manipulation. The most pronounced effects concerned perceptions of attraction grounded primarily in physical and sexual factors, as well as expectations regarding relational stability and trust. For instance, participants were more likely to endorse items suggesting that the partners based their relationship primarily on physical and sexual attraction (e.g., “They base their relationship on physical and sexual attraction”, or “They have a relationship that is more physical and sexual than emotional”), *F*_s_(2, 478) ≥ 3.91, *p*_s_ ≤ .021, *η_p_*^2^*_s_* ≥ .016. In addition, online-initiated relationships were more strongly associated with skepticism regarding future fidelity, as reflected in higher agreement with items implying potential infidelity (e.g., “They might cheat on each other in the future”), *F*(2, 478) = 6.90, *p* < .001, *η_p_*^2^ = .028, or the possibility that the partners were already engaged in a non-exclusive relationship (e.g., “They might have an open relationship”), *F*(2, 478) = 7.44, *p* < .001, *η_p_*^2^ = .030. Importantly, all items revealed a pattern of results consistent with those observed with the analyses of the entire scale of negative characterization: prejudice was directed toward couples who met online, regardless of whether the relationship originated on Facebook or Tinder, with both online contexts eliciting more negative evaluations of the couple if compared with the offline condition. Still, as these item-level analyses were multiple and exploratory, the observed effects should be considered with caution; future studies are therefore needed to confirm these preliminary insights through hypothesis-driven designs.

### 3.3. Discussion

Taken together, the findings of Study 2 sustain the existence of prejudice towards online-formed couples. First, although the origin manipulation did not influence positive characterization of the evaluated couple, exploratory item-level analyses revealed that partners who met online—and particularly those who met via Tinder—were perceived as less willing to disclose the circumstances of their encounter. This suggests the projection of anticipated embarrassment, as if online-formed couples are expected to feel a sense of social discomfort regarding the digital onset of their relationship.

Second, results also showed a clear effect of the origin condition on the negative characterization of the evaluated couple, with participants attributing systematically higher levels of negative characteristics to couples whose relationship originated online—whether on Facebook or Tinder. This pattern suggests that the primary driver of negative attribution is the digital nature of the first encounter, rather than the specific use of a dating app. Since both online origins appeared to trigger a degree of prejudice regarding a couple’s characteristics, the stigma seemed to be rooted in the broader online-mediated onset of the relationship.

## 4. General Discussion

The present research investigated the social stigma surrounding dating apps (DAs) like Tinder and, more generally, all forms of digital romantic encounters, moving from the evaluation of a single potential partner (Study 1) to that of an established couple (Study 2). Taken together, our findings confirm that a prejudice toward online dating, and Tinder in particular, *does exist* and appear to predict social perception at both the individual and relational levels—though its manifestation is more nuanced and slightly different from what we expected to be a simple, platform-specific “Tinder bias”.

As expected, Study 1 demonstrated that pre-existing prejudice toward Tinder was associated with less favorable impressions on a new potential partner met online: high-prejudice individuals appeared to spend less time processing Tinder profiles and attribute more negative traits to the target. Notably, this association emerged regardless of whether the profile was encountered on Facebook or Tinder. Furthermore, the study suggested that even users who had already used dating apps in the past might exhibit an interesting form of *internalized bias*, tending to report lower attraction toward the target when their profile was presented as coming from Tinder.

Study 2 shifted to the relational level, revealing that, as hypothesized, knowing that an established couple originated from an online dating—whether on Facebook or Tinder—significantly increased negative impressions on that couple. Specifically, online-initiated relationships were perceived as more physically and sex-oriented, and more prone to instability and infidelity than those who started offline. No effect was instead found on the attribution of positive characteristics, such as the presence of a sincere emotional bond and harmony. This suggests that while love within digital couples is not questioned, the bases of it, and the stability of such a partners’ emotional bond remain under judgment. Furthermore, a noteworthy result of Study 2 suggests the existence of a sort of “projected embarrassment”: participants assumed that online-formed couples would feel the need to hide their origin in social contexts, especially if they met on Tinder.

### 4.1. Tinder Prejudice as a Cognitive Shortcut

Most of the present findings are in line with what one would expect in light of literature on prejudice. The interaction between prejudice and profile source on attention dedicated to a potential partner’s profile, for instance, is potentially consistent with the cognitive miser perspective of Fiske and Taylor (Fiske & Taylor, 1984; Taylor & Fiske, 1978; for recent works and/or editions see also Fiske & Taylor, 2020a, 2020b). Their theorization suggests that individuals tend to minimize (and save) cognitive effort by relying on heuristics and pre-existing schemas, such as stereotypes. Our results suggested that participants with high prejudice toward Tinder indeed spent significantly less time examining the target’s profile when it was presented as coming from that platform compared to Facebook. In this sense, prejudice would function as a powerful cognitive shortcut: high-prejudice participants do not need to deeply process a new potential partner’s specific information (such as their biographical details or photographs) because their negative stereotype of the typical Tinder user already provides a ready-made impression. This top-down processing perhaps allows them to rapidly characterize the potential partner, leading to a premature closure of the information-gathering phase. Conversely, the allocation of more attention to a generic social media profile—showed in our study by high-prejudice participants, and among low-prejudice participants—suggests that, when a negative schema is not triggered, individuals are more likely to engage in a more nuanced, bottom-up evaluation of the potential partner’s profile. However, as the interaction on dwell time yielded a relatively small effect size, these findings should be considered preliminary, and further research is needed to confirm the robustness of this heuristic-based processing in the context of digital dating.

As expected, Study 1 suggested that pre-existing prejudice toward Tinder is associated with less favorable impressions of a new potential partner met online, with high-prejudice individuals attributing more negative traits to the target. Contrary to our initial hypotheses, however, this association emerged regardless of whether the profile was encountered on Facebook or Tinder. This potentially suggests that such prejudice may influence the perception of online romantic encounters more broadly, rather than being strictly platform-specific. Though, as Study 1 lacked an offline control condition, this interpretation remains exploratory; future research implementing a face-to-face baseline is necessary to definitively determine whether this negative trait attribution is uniquely triggered by the digital nature of the encounter.

Relatedly, it is possible that the specific task of evaluating a ‘potential partner’ led participants to associate Facebook with a dating context. Since the experimental framing was inherently romantic, the Facebook profile might have been interpreted through a dating-oriented lens, regardless of the platform’s general purpose. Study 2 reinforces this perspective, suggesting that once a romantic frame is established, the specific platform becomes secondary to the digital nature of the encounter. Future studies could address this by removing the romantic framing to see if the prejudice toward Tinder still generalizes to other digital platforms when the interaction is not aimed at relationship formation.

### 4.2. The User’s Paradox: Internalized Stigma and the “Tinder Gaze”

The interaction effect between past experience with DAs and profile source also offers a fascinating, albeit paradoxical, insight into the user’s perspective. While experienced users generally evaluate online-met potential partners more favorably, this openness seems to vanish when they encounter a profile specifically on Tinder. This result might be interpreted through the lens of context-dependent standards of attraction. It is possible that experienced users, being familiar with the swiping-based, visually driven nature of Tinder, unconsciously raise the bar for what they consider attractive within that specific environment. In the infinite scrolling of Tinder—where users are habituated to a constant stream of potential partners, all presenting highly curated photographs—a profile perceived as attractive enough on a general social network like Facebook might fail to meet the heightened aesthetic standards of the Tinder ecosystem. This suggests that a sort of Tinder gaze (i.e., a more evaluative “Tinder eye”) might be inherently more critical and demanding. Conversely, when the same profile is seen on Facebook, the target’s attractiveness may stand out more prominently.

According to another lecture, this reduction in attraction towards Tinder users, exhibited by people who are or have been users themselves in the past, might be a symptom of a “dating app fatigue” or a defensive mechanism. Experienced users, having navigated the disappointments of online dating, might develop a more cynical or “tired” gaze. Encountering the profile of yet another potential partner acts, in this sense, as a prime, reactivating the negative cognitive associations and emotional experiences that they associate with the use of Tinder. Such a scenario would be coherent with what Her and Timmermans (2021) would call the “Tinder blue” effect. Their research demonstrates that platform use is often associated with lower self-esteem and increased loneliness. For a user burdened by this effect, the target profile might be perceived through a disillusioned lens that colors the perception of that individual with the frustrations inherent to the dating app environment itself—a dynamic that may particularly apply to our participants, as they were all *single* with past DA experience.

A third and perhaps more theoretically grounded interpretation of this interaction involves social identity theory (SIT; Tajfel & Turner, 1979) and the phenomenon of in-group distancing (van Veelen et al., 2020). Our results show that the devaluation of Tinder profiles tended to specifically occur among participants with past DA experience. From a SIT perspective, when a social category is associated with negative stereotypes—such as being desperate or superficial—members of that group may engage in identity management strategies to protect their self-esteem (Tajfel & Turner, 1979). One such strategy is distancing oneself from the typical or prototypical in-group member. By evaluating a Tinder profile more critically, experienced users may be implicitly signaling that they do not belong to the stigmatized “mass” of users, thereby maintaining a positive social identity despite their own use of the platform. Conversely, for non-users, the Tinder label might not have triggered a similar shift in attraction because they lack a shared social identity to protect, resulting in a stable, albeit lower, baseline evaluation.

### 4.3. The Stigma of Origin and the Perceived Authenticity of Online-Formed Couples

The findings of Study 2 on prejudice towards online-formed couples also offer valuable insights. First of all, these results align with previous research and clearly suggest that a “stigma of origin” does really exists and does not merely affect the initial stages of dating but persists even after years of a stable, committed relationship (e.g., Wildermuth, 2004; Riger, 2017).

Interestingly, Study 2 revealed no significant differences in the perception of online-formed relationships between the Tinder and Facebook conditions. While we initially hypothesized a Tinder-specific stigma, these findings suggest that the observed prejudice might be rooted in the digitally mediated nature of the encounter itself, rather than the specific platform used—a possibility one can derive also from Study 1’s results. In other words, the ‘online’ origin of a relationship—regardless of whether it stems from a dating app or a general social network—seems to be the primary driver of the social stigma.

In this respect, it is worth noting that although Facebook has introduced dedicated dating features (i.e., Facebook Dating), this service remains marginal in the Italian context, with a penetration rate of only 3% (YouGov, 2021). Therefore, it is highly unlikely that our participants perceived the Facebook condition as an intentional dating-app encounter. Rather, the similarity in results between Tinder and Facebook, consistently found in both Studies 1 and 2, more likely reflects a generalized bias toward all forms of digitally initiated relationships, which are perceived as less ‘authentic’ or stable than those started offline. Future studies might be interested in further investigating this possibility.

Dating apps, in any case, are designed to *facilitate* social encounters. Therefore, prejudice toward online dating—especially when extended to neutral social platforms like Facebook—risks obstructing the potential benefits for individuals who struggle to form connections in their everyday lives. In this context, a dating app should be viewed as one instrument among many to expand social opportunities, allowing users to meet uncommitted individuals and potentially establish stable relationships, much like traditional offline pathways. Qualitative work by Machfudz et al. (2021), for instance, demonstrates that online matches can progress toward deep emotional intimacy, commitment, and even marriage, following recognizable relationship-development patterns.

### 4.4. The Reality of Digital Relationships: Challenging Stereotypes Through Emotional Intensity

The benefits of using dating apps are documented in past research, with some studies even suggesting that online-initiated relationships may possess certain advantages over offline ones. Potarca (2020), for instance, found that couples formed through mobile dating apps often report stronger intentions to cohabit than those formed in non-digital settings. Furthermore, women who met their partners through a dating app appear to express stronger fertility desires and intentions compared to those who established their relationships offline.

Contrary to the negative stereotypes of superficiality and instability observed in our studies, another recent qualitative evidence from Sharabi (2023) suggests that meeting online may even foster a unique road to marriage. In her study of married and engaged couples who met via dating apps, Sharabi identified several positive long-term trajectories. Online platforms appear to facilitate a process of technology-enabled relationship initiation that emphasizes compatibility and intentionality from the very beginning. Because users often enter these spaces with the explicit goal of finding a partner, they engage in more direct communication regarding their values and future expectations. Also, these couples often experience multimodal development, where technology continues to reinforce their bond even after transitioning offline. Finally, Sharabi identifies outcomes that contradict common prejudices: online-met couples often report high levels of marital success, rooted in the breakdown of traditional social barriers and a more deliberate approach to courtship.

There is also a theoretical basis to suggest that online-formed couples might develop stronger emotional bonds and more stable relationships when facing adversity. According to Brehm’s (1999) emotional intensity theory (EIT), the presence of an obstacle can actually augment romantic feelings. A growing body of experimental research supports this hypothesis, demonstrating that when a couple is forced to confront external challenges—such as the stress stemming from social stigma and prejudice often observed in devaluated or marginalized relationships (e.g., same sex, interracial, or age-gap relationships, Lehmiller & Agnew, 2006)—these obstacles can intensify their emotional connection and ultimately strengthen the relationship (Sciara & Pantaleo, 2018; Donato et al., 2018; Miron et al., 2009; for a review, see Sciara & Pantaleo, 2023). This specific dynamic is historically referred to as the “Romeo and Juliet effect” (Driscoll, 2014; Driscoll et al., 1972) and could be highly congruent with Sharabi’s (2023) findings. It is possible that navigating social prejudice against dating apps acts as an initial bond-strengthening obstacle, encouraging the deliberate and intentional courtship observed in her study. In this sense, the theoretical perspective offered by EIT might explain why some online-met couples develop resilient long-term trajectories, providing a promising avenue for future research on how stigma paradoxically reinforces relationship stability.

When the research findings discussed above are considered alongside our results, a striking paradox emerges: while research demonstrates that digital onsets can lead to stable, highly committed, and high-quality relationships, the public still perceives these couples through a lens of skepticism and projected embarrassment. Ultimately, maintaining a pervasive prejudice toward these platforms risks precluding individuals from accessing these significant relational and social opportunities.

It is important to note that documenting the persistence of prejudice towards online dating does not imply that dating apps are free from objective risks and negative effects. The skepticism observed in our participants may be partly rooted in a social awareness of real challenges that dating apps pose, such as the frequent expectancy violations one may experience when switching from digital to face-to-face dating (e.g., Sciara et al., 2021), the detrimental impact that daily use of dating apps generally has on users’ self-esteem—including increased loneliness and psychological distress (e.g., Her & Timmermans, 2021)—or the potential risk of developing an addiction (e.g., Thomas et al., 2025; Vera Cruz et al., 2024). When reality fails to match the idealized online image, relationships are often expected to end prematurely or never initiate at all. In other cases, when the use of a dating app becomes too frequent in daily life, it can act as a time-consuming endeavor that diverts attention away from traditional social interactions and translates into a true addiction. Some people, research shows, even use DAs to escape from other problems that occupy their everyday life (Thomas et al., 2025). While people should be aware of all these negative effects, our findings suggest that this realistic concern often hardens into a rigid prejudice that overlooks the many couples who successfully navigate these hurdles (e.g., Sharabi, 2023; Rosenfeld, 2017). Ultimately, the challenge is to distinguish between a legitimate recognition of dating app pitfalls—with, of course, subsequent efforts to prevent and/or resolve them—and the derogatory stereotypes that unfairly penalize digital bonds, even in the most positive cases.

### 4.5. Limitations and Future Directions

Despite the insights provided, the present research has limitations. First, Study 1 compared two digital contexts (Facebook vs. Tinder) without including a traditional offline control condition. While this choice was dictated by the methodological need to keep visual and textual stimuli constant across groups—an operation that would be extremely difficult when transitioning from digital to face-to-face settings—future research should aim to develop creative methodologies to allow for such a direct comparison.

Second, the “potential partner” framing used in Study 1 may have primed high-prejudice individuals, activating negative schemas. Future studies should employ a more neutral frame (e.g., presenting targets simply as “individuals” or “acquaintances”) to avoid any explicit reference to romantic dating and thus determine if this prejudice persists outside of romantic contexts.

Finally, it should be noted that the positive traits scale used in Study 1 showed low reliability (*α* = 0.60). This lower internal consistency may stem from the inherent breadth of the questionnaire created ad hoc, which aimed to capture a wide array of desirable personal qualities, combined with the limited number of items used (5 items). Future studies should employ more specialized and extensive scales to assess personality traits perception with greater precision.

Some limitations also pertain to our Study 2 on the perception of established relationships initiated via an online encounter, with some questions regarding the long-term perception of online-initiated couples remaining open. A primary limitation and an intriguing direction for future studies concerns the perceived and actual longevity of these relationships. While our findings highlight a clear prejudice toward the stability of online-formed couples, we did not directly measure whether this bias translates into a specific lower estimation of relationship duration compared to offline encounters.

Regarding both the experiments, it should be noted that while the samples’ age range closely reflected the primary demographic of Tinder users (Iovino, 2026), the reliance on convenience, social media, and snowball sampling among young adults may limit the immediate generalizability of the present results to older cohorts or different cultural contexts. In this regard, further studies are needed to explore how prejudice in older populations shapes their evaluation of dating app users and digital initiation, as different generations may hold distinct social norms regarding relationship formation.

Future research should also explore the effects of DAs prejudice by adopting a more theoretically driven approach, drawing for instance on social identity theory (SIT) as a robust explanatory framework. As our findings suggest, SIT—and specifically the mechanism of in-group distancing—offers a compelling explanation for the paradoxical devaluation of Tinder profiles observed among experienced users. Building on this, future experimental designs could directly manipulate the salience of social identity or investigate the role of internalized stigma in shaping interpersonal evaluations.

Also, the literature currently lacks comprehensive longitudinal data and experimental evidence directly comparing the estimated vs. actual survival rates of relationships across these different onset contexts. While some studies suggest that online dating may, in fact, favor stability due to greater compatibility matching (e.g., Cacioppo et al., 2013; Potarca, 2020; Rosenfeld, 2017), others have pointed to a “choice overload” effect that might undermine long-term commitment (e.g., D’Angelo & Toma, 2017). Future research should therefore specifically investigate whether the negative characterization observed here translates into shorter relationship duration. Understanding whether online-formed couples actually face a higher risk of dissolution—or if they are simply victims of a persistent social stigma that underestimates their endurance—would be a crucial step toward deconstructing this stigma.

In conclusion, although this research acknowledges certain limitations, the findings provide a critical foundation for better understanding the enduring prejudice toward dating apps—and online dating in general—in modern social perception. These results open new avenues for future interventions aimed at normalizing digital romantic experiences and reducing the social costs of meeting online. Ultimately, in an era where our lives are inextricably linked to technology and the digital world, moving beyond this digital stigma will be essential to ensure that individuals can fully embrace the relational opportunities offered by technology without the fear of social devaluation.

## Figures and Tables

**Table 1:** Means and Standard Deviations for Time Spent Viewing the Profile, Attraction, and Positive and Negative Trait Attribution as a Function of Prejudice toward Tinder and Profile Source (Study 1).

	Low Prejudice		High Prejudice	
	Facebook	Tinder	Facebook	Tinder
	*n* = 54	*n* = 53	*n* = 46	*n* = 53
Dedicated Time (s)	30.44 (13.18)	31.96 (21.66)	37.85 (27.94)	27.75 (19.51)
Attraction	6.42 (1.56)	5.90 (1.63)	5.96 (2.16)	6.00 (1.93)
Positive Traits	3.69 (0.47)	3.67 (0.37)	3.68 (0.45)	3.63 (0.41)
Negative Traits	2.33 (0.55)	2.46 (0.48)	2.51 (0.44)	2.57 (0.54)

*Note.* Values represent means, with standard deviations in parentheses; *n_s_* refer to numerosity for each experimental condition (*N* = 206). Time dedicated to the profile is expressed in seconds; interpersonal attraction was measured on an 11-point scale; attribution of positive and negative traits was measured on a 5-point scale.

**Table 2:** Means and Standard Deviations for Positive and Negative Characterization of the Evaluated Couple (Study 2).

	Origin of the Couple		
	Offline	Facebook	Tinder
	*n* = 173	*n* = 148	*n* = 160
Positive Characterization	6.35 (0.92)	6.26 (0.95)	6.22 (0.95)
Negative Characterization	4.65 (1.21)	5.13 (1.19)	5.30 (1.23)

*Note.* Values represent means, with standard deviations in parentheses; *n_s_* refer to numerosity for each experimental condition (*N* = 481). Positive and negative characterizations of the target couple were measured using a 22-item questionnaire focused on both positive (e.g., emotional bond and harmony) and negative characteristics (e.g., signs of potential relational instability). All items were scored on a 9-point Likert-like scale.

## Data Availability

All studies’ data are available on Open Science Framework (OSF) at https://osf.io/ze3s5/overview?view_only=f4cb91bf2e8240d6a0ddf5d5ad968107. The authors are also willing to share their analytics methods and studies materials with other researchers upon request.

## Supplementary Materials

The following supporting information can be downloaded at: https://www.mdpi.com/article/10.3390/bs16050691/s1, Supplementary Materials, Section S1: Experimental Manipulation and Detailed Instructions of Study 1; and Section S2: Experimental Manipulation and Detailed Instructions of Study 2.

## References

[B1-behavsci-16-00691] Anderson T. L. (2005). Relationships among internet attitudes, internet use, romantic reliefs, and perceptions of online romantic relationships. CyberPsychology and Behavior.

[B2-behavsci-16-00691] Biernat M., Vescio T. K., Green M. L. (1996). Selective self-stereotyping. Journal of Personality and Social Psychology.

[B3-behavsci-16-00691] Brehm J. W. (1999). The intensity of emotion. Personality and Social Psychology Review.

[B4-behavsci-16-00691] Cacioppo J. T., Cacioppo S., Gonzaga G. C., Ogburn E. L., VanderWeele T. J. (2013). Marital satisfaction and break-ups differ across on-line and off-line meeting venues. Proceedings of the National Academy of Sciences.

[B5-behavsci-16-00691] D’Angelo J. D., Toma C. L. (2017). There are plenty of fish in the sea: The effects of choice overload and reversibility on online Daters’ satisfaction with selected partners. Media Psychology.

[B6-behavsci-16-00691] Degen J., Kleeberg-Niepage A. (2022). The more we Tinder: Subjects, selves and society. Human Arenas.

[B7-behavsci-16-00691] Donato S., Parise M., Pagani A. F., Sciara S., Iafrate R., Pantaleo G. (2018). The paradoxical influence of stress on the intensity of romantic feelings towards the partner. Interpersona: An International Journal on Personal Relationships.

[B8-behavsci-16-00691] Donn J. E., Sherman R. C. (2002). Attitudes and practices regarding the formation of romantic relationships on the internet. CyberPsychology & Behavior.

[B9-behavsci-16-00691] Driscoll R. (2014). Commentary and rejoinder on Sinclair, Hood, and Wright (2014): Romeo and Juliet through a narrow window. Social Psychology.

[B10-behavsci-16-00691] Driscoll R., Davis K. E., Lipetz M. E. (1972). Parental interference and romantic love: The Romeo and Juliet effect. Journal of Personality and Social Psychology.

[B11-behavsci-16-00691] Duguay S. (2017). Dressing up Tinderella: Interrogating authenticity claims on the mobile dating app Tinder. Information, Communication & Society.

[B12-behavsci-16-00691] Esposito R. (2024). Uso di Facebook in Italia: Dati aggiornati.

[B13-behavsci-16-00691] Fang W. Q., Lee Y. C., Rau P. L. P. (2023). Effect of editing photos by application on Chinese facial impression perception. Cross-cultural design.

[B14-behavsci-16-00691] Faul F., Erdfelder E., Lang A.-G., Buchner A. (2007). G*Power 3: A flexible statistical power analysis program for the social, behavioral, and biomedical sciences. Behavior Research Methods.

[B15-behavsci-16-00691] Finkel E. J., Eastwick P. W., Karney B. R., Reis H. T., Sprecher S. (2012). Online dating: A critical analysis from the perspective of psychological science. Psychological Science in the Public Interest.

[B16-behavsci-16-00691] Fisher H. E., Garcia J. R., Sternberg R. J., Weis K. (2019). Slow love: Courtship in the digital age. The new psychology of love.

[B17-behavsci-16-00691] Fiske S. T., Taylor S. E. (1984). Social cognition.

[B18-behavsci-16-00691] Fiske S. T., Taylor S. E. (2020a). Social cognition evolves: Illustrations from our work on intergroup bias and on healthy adaptation. Psicothema.

[B19-behavsci-16-00691] Fiske S. T., Taylor S. E. (2020b). Social cognition: From brains to culture.

[B20-behavsci-16-00691] Gatter K., Hodkinson K. (2016). On the differences between Tinder versus online dating agencies: Questioning a myth. An exploratory study. Cogent Psychology.

[B21-behavsci-16-00691] Hancock J. T., Toma C. L. (2009). Putting your best face forward: The accuracy of online dating photographs. Journal of Communication.

[B22-behavsci-16-00691] Her Y.-C., Timmermans E. (2021). Tinder blue, mental flu? Exploring the associations between Tinder use and well-being. Information, Communication & Society.

[B23-behavsci-16-00691] Holland G., Tiggemann M. (2016). A systematic review of the impact of the use of social networking sites on body image and disordered eating outcomes. Body Image.

[B24-behavsci-16-00691] Iovino C. (2026). Statistiche Tinder 2026—Utenti e ricavi (dati globali). *Cristian Iovino Blog*.

[B25-behavsci-16-00691] Johanis T. C., Midgley C. E., Lockwood P. (2024). Desperate or desirable? Perceptions of individuals seeking dates online and offline. Personal Relationships.

[B26-behavsci-16-00691] Krüger S., Spilde A. C. (2020). Judging books by their covers—Tinder interface, usage and sociocultural implications. Information, Communication & Society.

[B27-behavsci-16-00691] Latrofa M., Vaes J., Cadinu M., Carnaghi A. (2010). The cognitive representation of self-stereotyping. Personality and Social Psychology Bulletin.

[B28-behavsci-16-00691] Lehmiller J. J., Agnew C. R. (2006). Marginalized relationships: The impact of social disapproval on romantic relationship commitment. Personality and Social Psychology Bulletin.

[B29-behavsci-16-00691] Machfudz F. S., Boer R. F., Wongso N. (2021). Cyberintimacy involvement on building emotional intimacy in close relationship on Indonesian Tinder users. Jurnal Komunikasi Ikatan Sarjana Komunikasi Indonesia.

[B30-behavsci-16-00691] Martinovic B., Verkuyten M. (2013). ‘We were here first, so we determine the rules of the game’: Autochthony and prejudice towards out-groups. European Journal of Social Psychology.

[B31-behavsci-16-00691] Miron A. M., Knepfel D., Parkinson S. K. (2009). The surprising effect of partner flaws and qualities on romantic affect. Motivation and Emotion.

[B32-behavsci-16-00691] Parsa K. M., Charipova K., Chu E., Reilly M. J. (2022). Social perception of self-enhanced photographs. Facial Plastic Surgery.

[B33-behavsci-16-00691] Potarca G. (2020). The demography of swiping right: An overview of couples who met through dating apps in Switzerland. PLoS ONE.

[B34-behavsci-16-00691] Ranzini G., Lutz C. (2017). Love at first swipe? Explaining Tinder self-presentation and motives. Mobile Media & Communication.

[B35-behavsci-16-00691] Riger D. (2017). Perceptions of stigma in online dating narratives: Implications for marriage and family therapists. Doctoral dissertation.

[B36-behavsci-16-00691] Rosenfeld M. J. (2017). Marriage, choice, and couplehood in the age of the internet. Sociological Science.

[B37-behavsci-16-00691] Rosenfeld M. J., Thomas R. J., Hausen S. (2019). Disintermediating friends: How online dating in the United States overcomes traditional role of social networks. Proceedings of the National Academy of Sciences.

[B38-behavsci-16-00691] Sawyer A., Smith E., Benotsch E. (2018). Dating application use and sexual risk behavior among young adults. Sexuality Research and Social Policy.

[B39-behavsci-16-00691] Sciara S., Malighetti C., Martini G., Riva G., Regalia C. (2021). Idealization on dating apps: Seeing fewer photos of the potential partner leads to expectancy violation and lower attraction. Annual Review of CyberTherapy and Telemedicine.

[B40-behavsci-16-00691] Sciara S., Pantaleo G. (2018). Relationships at risk: How the perceived risk of ending a romantic relationship influences the intensity of romantic affect and relationship commitment. Motivation and Emotion.

[B41-behavsci-16-00691] Sciara S., Pantaleo G., Mogislki J., Shackelford T. (2023). In-pair divestment. The Oxford handbook of evolutionary psychology and romantic relationships.

[B42-behavsci-16-00691] Sharabi L. L. (2023). The enduring effect of internet dating: Meeting online and the road to marriage. Communication Research.

[B43-behavsci-16-00691] Silva R. R., Koch M.-L., Rickers K., Kreuzer G., Topolinski S. (2019). The Tinder™ stamp: Perceived trustworthiness of online daters and its persistence in neutral contexts. Computers in Human Behavior.

[B44-behavsci-16-00691] Sprecher S., Schwartz P., Harvey J., Hatfield E., Sprecher S., Wenzel A., Harvey J. (2008). TheBusinessofLove.com: Relationship initiation at internet matchmaking services. Handbook of relationship initiation.

[B45-behavsci-16-00691] Strubel J., Petrie T. A. (2017). Love me Tinder: Body image and psychosocial functioning among men and women. Body Image.

[B46-behavsci-16-00691] Sumter S. R., Vandenbosch L., Ligtenberg L. (2017). Love me Tinder: Untangling emerging adults’ motivations for using the dating application Tinder. Telematics and Informatics.

[B47-behavsci-16-00691] Tajfel H., Turner J. C., Austin W. G., Worchel S. (1979). An integrative theory of intergroup conflict. The social psychology of intergroup relations.

[B48-behavsci-16-00691] Taylor S., Fiske S. (1978). Salience, attention, and attribution: Top of the head phenomena. Advances in Experimental Social Psychology.

[B49-behavsci-16-00691] Thomas M. F., Dörfler S., Mittmann G., Steiner-Hofbauer V. (2025). Problematic online dating: Systematic review of definitions, correlates, and study designs. Journal of Medical Internet Research.

[B50-behavsci-16-00691] Timmermans E., Courtois C. (2018). From swiping to casual sex and/or committed relationships: Exploring the experiences of Tinder users. The Information Society.

[B51-behavsci-16-00691] Valkenburg P. M., Peter J. (2007). Who visits online dating sites? Exploring some characteristics of online daters. CyberPsychology & Behavior.

[B52-behavsci-16-00691] van Veelen R., Veldman J., van Laar C., Derks B. (2020). Distancing from a stigmatized social identity: State of the art and future research agenda on self-group distancing. European Journal of Social Psychology.

[B53-behavsci-16-00691] Vera Cruz G., Aboujaoude E., Rochat L., Bianchi-Demicheli F., Khazaal Y. (2024). Online dating: Predictors of problematic Tinder use. BMC Psychology.

[B54-behavsci-16-00691] von Hippel W., Sekaquaptewa D., Vargas P. (1997). The linguistic intergroup bias as an implicit indicator of prejudice. Journal of Experimental Social Psychology.

[B55-behavsci-16-00691] West K., Hotchin V., Wood C. (2017). Imagined contact can be more effective for participants with stronger initial prejudices. Journal of Applied Social Psychology.

[B56-behavsci-16-00691] Wildermuth S. M. (2004). The effects of stigmatizing discourse on the quality of on-line relationships. CyberPsychology & Behavior.

[B57-behavsci-16-00691] YouGov (2021). La diffusione delle app di incontri in Italia.

