# Supplementary material for: Exploring Prejudice Toward Tinder: Two Experiments on the Social Perception of Dating App Users and Online-Formed Couples
Source: Behav Sci (Basel). 2026 Apr 30;16(5):691. doi: 10.3390/bs16050691 (PMC13203467; doi:10.3390/bs16050691)
Supplement: Supplementary file 1 [file behavsci-16-00691-s001.zip › behavsci-4197824-supplementary.pdf]

Supplementary Materials for the article "Exploring Prejudice Toward Tinder: Two Experiments on the Social Perception of Dating App Users and Online-Formed Couples"

Simona Sciara, Federico Contu, Federica Montano, Carolina Sole Steffano, Giuseppe Pantaleo

S1. Experimental Manipulation and Detailed Instructions of Study 1

Participants of Study 1 were randomly assigned to evaluate a potential partner's profile, originating from either Facebook (control) or Tinder (experimental). To ensure internal validity, visual and textual stimuli were identical across conditions: all participants viewed the same set of six photographs and biographical description. The only variation was the branding identifying the source and the text presenting the profile. Crucially, the platform was mentioned without additional framing to avoid biasing the perception of Facebook in a romantic key.

The specific instructions provided to participants were as follows (translated from Italian):

"A Tinder [Facebook] user has consented to the use of the images from their profile for research purposes. In this first part of the questionnaire, we will therefore ask you to observe his/her profile (all 6 images posted by him/her + his/her bio), form an impression, and then answer some questions. Please, take all the time you need to understand if you might like this person."

The biographical text provided to participants, identical for both male and female targets, was as follows (translated from Italian):

"Hi! My name is Marco [Alice] and I live in Milan. I moved here to study economics and I now work for an insurance company. Although I like Milan very much, I miss the sea in Bari, where I was born and raised. I am a lover of good food, an adventurer, and a traveler. With just a backpack on my shoulders, I would go anywhere!"

S2. Experimental Manipulation and Detailed Instructions of Study 2

Participants were randomly assigned to one of three conditions (offline, Facebook, or Tinder) to read a standardized description of a couple's meeting. The manipulation was limited to the encounter's setting, keeping all other biographical and narrative elements constant to ensure internal validity. Specifically, participants were presented with the following prompt (translated from Italian):

"You will now be shown some photos of a real-life couple who have given their consent for the use of their photos, names, and story for research purposes. This is Giulia, 21, and Marco, 24. They met a few months ago on Tinder [or Facebook / at a bar], an app [a social media / a bar] both had been using [attending] for some time. Marco was about to complete his degree in biology and Giulia had recently finished high school. Observe their photos for as long as you need. Then answer the following questions based on the impression you have formed of Giulia and Marco and their relationship."
